# Public Cognizance Concerning Skin Cancer in the Qassim Region of Saudi Arabia: A Cross-Sectional Study

**DOI:** 10.7759/cureus.108673

**Published:** 2026-05-11

**Authors:** Amal Ismail, Reema Albreah, Shoug Alqarawi, Lama AlAsqah, Worod Almutairi, Waad Alharbi, Kadi Al Shibl, Rawan Alrumaih

**Affiliations:** 1 Pharmacy Practice Department, College of Pharmacy, Qassim University, Unaizah, SAU; 2 Pharmacy Practice Department, Qassim University, Unaizah, SAU

**Keywords:** attitude, knowledge, prevention, qassim, saudi arabia, skin cancer, sun protection

## Abstract

Background

Skin cancer is a growing public health concern in Saudi Arabia, with ultraviolet (UV) radiation recognized as the primary modifiable risk factor. Public awareness and preventive behaviours are essential for early detection. This study assessed knowledge, attitudes, and risk‑related characteristics concerning skin cancer among adults in the Qassim region.

Methods

A descriptive cross‑sectional study was conducted among adults (≥18 years) residing in Qassim. Data were collected over three months using a validated, self‑administered online questionnaire distributed via social media platforms. The survey assessed demographics, phenotypic risk factors, knowledge, and attitudes. Data were analysed using SPSS version 25 (IBM Corp., Armonk, NY), employing descriptive statistics and chi‑square tests to examine associations between sociodemographic variables and knowledge categories.

Results

A total of 3,420 participants were included; 60.5% were female participants and 94.4% were younger than 50 years. Most respondents (58.5%) exhibited darker phenotypic traits, and 69.8% reported having fewer than five moles. Overall, 74.9% demonstrated good knowledge of skin cancer, and 94.1% expressed positive attitudes towards prevention. Educational level showed a statistically significant association with knowledge status (χ² = 34.723, p < 0.001), indicating higher knowledge among participants with university or postgraduate education. Age was also significantly associated with awareness (p < 0.001).

Conclusion

Participants demonstrated generally good knowledge and positive attitudes towards skin cancer prevention despite belonging to a predominantly lower‑risk demographic. However, substantial gaps persisted regarding hereditary risk, mucosal involvement, and recognition of chronic non‑healing wounds. Targeted educational interventions, particularly for individuals with lower educational attainment, are recommended to strengthen early detection practices and promote comprehensive sun‑protective behaviours.

## Introduction

Skin cancer is the most common type of cancer worldwide and the ninth most common malignancy in Saudi Arabia. The rate of occurrence is apparently highest in lighter-skinned individuals and is much rarer in darker-skinned ones. Significant geographical and racial distinctions have been reported. Caucasian ethnicity has been described as being most frequently involved in skin cancer, with a very high occurrence in specific countries such as Australia and New Zealand, which have been recorded as the areas of maximum incidence, followed by the southern areas of America inhabited by a White population. On the other hand, in Southeastern and South-Central Asia, the Asian population showed lower incidence rates (below 0.5 per 100,000) in both men and women [1,2].

Studies from Saudi Arabia showed variations in the incidence of skin cancer types. In the Al-Taif region, the incidence of skin cancer has been documented among individuals aged 60 and above, exhibiting a male-to-female ratio of 2.25:1. Basal cell carcinoma (BCC) and squamous cell carcinoma (SCC) emerged as the most prevalent forms, accounting for 51% and 26%, respectively. In the Al-Baha region, located in the southwestern part of the Kingdom of Saudi Arabia, the mean age of skin cancer patients ranged from 70 to 80 years (with a male-to-female ratio of 1.6:1), where BCC, SCC, and Kaposi sarcoma (KS) were the predominant types, representing 41%, 29%, and 18%, respectively [3,4]. 

In Jeddah, which is located in the western region of the Kingdom, the average age of skin cancer patients was reported to be 46 years (with a male-to-female ratio of 2.1:1), with BCC, mycosis fungoides (MF), malignant melanoma (MM), and dermatofibrosarcoma protuberans (DFSP) being the most frequently observed malignancies, comprising 28.3%, 24.5%, and 10.3%, respectively [5]. Comparable findings were also noted in the Al Qassim region, located in the central part of the Kingdom [6]. Conversely, in the Dammam area, located in the eastern region of the Kingdom, BCC and SCC were identified as the most prevalent forms of skin cancer, with incidence rates of 26% and 22.2%, respectively [5].

Skin cancer subtypes include adnexal tumors like BCC, epidermal tumors like SCC, and melanocytic tumors like melanoma [7]. Skin cancer presents with varying clinical presentations that need a skin biopsy for histopathological examination and diagnosis necessary for therapy selection, such as local therapies (e.g., antineoplastic injections), systemic medications like chemotherapy and immunotherapy, radiation therapy, and surgical removal by Mohs micrographic surgery and cryosurgery [8,9].

Skin cancer is an interaction of variable genetic, phenotypic, and environmental factors. Increased risk of skin cancer includes individuals with a low Fitzpatrick phototype, in whom the patients have red hair and freckles. These populations have mutations in the melanocortin 1 receptor gene (*MC1*) affecting their ability to form melanin. The aetiology of melanoma is multifaceted and arises from the intricate interplay of genetic, phenotypic, and environmental determinants. Genetic susceptibility to melanoma arises from a multifaceted interplay of genes with varying levels of penetrance (high, moderate, and low), each influencing an individual’s likelihood of developing the disease. The degree of penetrance can also vary according to melanoma incidence across different populations and geographic regions. Advances in sequencing technologies have enabled the identification of additional genes implicated in melanoma and improved characterization of relevant germline variants. Although cyclin‑dependent kinase inhibitor 2A (*CDKN2A*) remains the most extensively studied gene associated with melanoma susceptibility, growing evidence indicates that several non‑CDKN2A genes also contribute meaningfully to risk. High‑penetrance genes include cyclin‑dependent kinase 4 (*CDK4*), BRCA1‑associated protein 1 (*BAP1*), protection of telomeres 1 (*POT1*), telomerase reverse transcriptase (*TERT*), adrenocortical dysplasia protein homolog (*ACD*), and telomeric repeat‑binding factor 2‑interacting protein (*TERF2IP*). Moderate‑penetrance genes consist of melanocortin 1 receptor (*MC1R*), microphthalmia‑associated transcription factor (*MITF*), and solute carrier family 45 member 2 (*SLC45A2*). Low‑penetrance variants include the oculocutaneous albinism type 2 gene (*OCA2*), tyrosinase‑related protein 1 (*TYRP1*), and tyrosinase (*TYR*). In addition to increasing melanoma susceptibility, some of these genetic alterations may also elevate the risk of developing other internal malignancies [10]. UV light exposure can cause damage to the skin's DNA, which requires repair. The ability to repair damage caused by UV light is defective in xeroderma pigmentosa, which is a DNA-repairing defect that predisposes to an increase in freckling. UV radiation exposure is considered the main risk factor, in addition to the presence of more than 50 naevi or having naevi showing dysplastic changes. Heavy metal exposure, like arsenic, and viral infections, such as human papillomavirus (HPV), play important roles in increasing the risk for skin cancer. Fair skin and increased exposure to ultraviolet B (UVB) rays were among the most significant risk factors. Fewer cases were seen among individuals with darker skin due to increased epidermal melanin, which provides greater photoprotection [9,11]. In the Qassim region, skin cancer is not uncommon. According to the literature, the most common malignant skin lesion was BCC (58.5%), followed by SCC (18.1%) [6].

The World Health Organization (WHO) has suggested several protective approaches to avoid the harmful effects of disproportionate exposure to UV rays, including social education and adopting positive behavioural changes [12].

The protection against skin cancer depends on knowledge about the preventive measures, and cancer prevention campaigns are targeting sun-related behaviour [2]. The level of knowledge, attitude, and behaviours is influenced by several factors such as age, education level, and skin colour. In the Qassim region, people believe that exposure to the sun is good for vitamin D, which is a common problem [13].

Skin cancer is often overlooked as a public health concern and tends to be underestimated. Therefore, assessing the level of public awareness is essential before developing effective prevention strategies [14]. Sunscreen is one of the most commonly used methods of photoprotection, and educational programmes have been shown to enhance knowledge and attitudes regarding the harmful effects of UVB radiation. Despite this, awareness remains limited in many smaller or underserved communities [15]. Importantly, skin cancer is largely preventable through health promotion and early detection. When melanoma is identified at an early stage, survival exceeds 98%, compared with roughly 15% for those diagnosed at an advanced stage. This highlights the need for healthcare providers to advocate for preventive measures and implement programmes that evaluate public awareness and emphasize reducing modifiable risk factors, particularly excessive sun exposure. Evidence also indicates that every unit of funding invested in sun‑safety education yields nearly fourfold savings in healthcare costs while reducing both morbidity and mortality associated with skin cancer [13].

Despite the increased incidence of skin cancer globally, there exists a notable deficiency of empirical evidence pertaining to the Saudi populace, particularly within the Qassim region. To remedy this shortfall, it is imperative to assess the levels of awareness, knowledge, and preventive behaviours, encompassing sun protection practices [16].

In Saudi Arabia, men don the thoub, an ankle-length garment that serves as a quintessential element of traditional male attire, while women adorn themselves in the abaya, a customary garment that envelops the entire body, leaving only the head, feet, and hands exposed. These forms of sun protection have demonstrated efficacy that is equal to, or even surpasses, that of conventional sunblock in mitigating the risk of sunburn. Numerous studies have been conducted in various countries to assess the knowledge, attitudes, and practices (KAP) of the public toward sun exposure and sun-protection measures, also for various sections of the general population, and thereby promote the perception of skin self-examination for early detection of skin cancers. The level of knowledge of the general population affects their attitude and their level of exposure to risk factors of skin cancer [17].

The study aimed primarily to assess the level of knowledge and attitudes towards skin cancer and its prevention among adults in the Qassim region of Saudi Arabia. The secondary objectives were to determine the prevalence of major phenotypic and behavioural risk factors for skin cancer; to examine the association between sociodemographic characteristics, particularly age and educational level, and knowledge about skin cancer; and to identify specific gaps in awareness related to hereditary risk, mucosal involvement, and recognition of chronic non‑healing wounds as early warning signs.

## Materials and methods

A descriptive quantitative cross-sectional study was conducted among the general population residing in the Qassim region, who are more than 18 years old and possess the minimal skills to participate in the electronic questionnaire that we published through public posts, community groups, university networks, social media, and emails over a period of three months between October 2022 and February 2023. No paid advertisements or targeted boosting were used; instead, recruitment relied on organic sharing and voluntary participation. To reduce duplicate responses, the survey platform restricted multiple submissions from the same device. Because the survey link was distributed through open social media channels and public community groups, the platform did not record the number of unique link views or click‑throughs. Therefore, a conventional response rate could not be calculated. All participants who accessed the survey and completed the mandatory items were included in the analysis. Incomplete questionnaires were excluded using listwise deletion.

This study was conducted in accordance with the ethical standards of the Declaration of Helsinki. Ethical approval was obtained from the Qassim Region Research Ethics Committee prior to data collection (Approval No. 200308). Participation was voluntary, and informed consent was obtained electronically from all respondents. No identifying information was collected, ensuring participant confidentiality and anonymity.

The self-administered questionnaire comprises four sections. The first section identifies the demographics and general criteria of the respondents. The second section consists of four questions that assess the risk of skin cancer among the participating population. Questions in the third section were formulated to evaluate the knowledge about skin cancer. The last section is composed of five Likert-scale-type questions to assess attitudes towards skin cancer and skin cancer prevention. (See the full version of the questions in the Appendix section.) The questionnaire was adopted and modified from the literature from previously validated study questionnaires [17-19] and revised and validated by two experts in the field of pathology and dermatology. Questionnaire questions were forward- and backward-translated. A pilot study was conducted to assess the questionnaire, and its internal consistency was evaluated using Cronbach’s alpha, which demonstrated good reliability $\left( \alpha=0.80 \right)$. Sample size was calculated using Raosoft sample size calculator, at a 5% margin of accepted error and a 95% confidence interval using the formula $x=Z\left( c/100 \right)2r\left( 100-r \right)$, $n=Nx/\left( \left( N-1 \right)E2+x \right)$, $E=\sqrt{ \left[ \left( N-n \right)x/n\left( N-1 \right) \right]}$, where N is the population size, r is the fraction of responses that are interested, and $Z\left( c/100 \right)$ is the critical value for the confidence level c. The representative sample size for the "1215858" residences in the Qassim region, according to the statistics, was 385 (version 2.3; Raosoft Inc., Seattle, WA, USA).

A convenient sampling method was followed in data collection. 

Data were entered into an Excel sheet (Excel, Microsoft Corporation, Redmond, WA, USA), coded, and exported to SPSS (Version 25, IBM Corp., Armonk, NY, USA) for further processing and statistical testing. Frequencies, cross-tabulation, and correlation were run as appropriate.

The knowledge score was calculated for the yes and no questions, and participants were classified accordingly into good knowledge and poor knowledge. Patients were classified into positive and negative attitudes according to their positive and negative scoring of attitude questions.

Knowledge scores were calculated by assigning one point for each correct response across the eight knowledge items, yielding a total possible score of 0-8. Consistent with prior community‑based cancer awareness studies, we classified scores <50% as ‘poor knowledge’ and ≥50% as ‘good knowledge,’ a threshold commonly used to enhance interpretability and comparability across populations. These cut-offs were also supported by the distribution of scores in our dataset, which demonstrated natural clustering around these intervals with minimal overlap between categories. Attitude scores were similarly categorized based on the proportion of positive responses across the Likert‑scale items.

Associations between categorical variables (e.g., education level vs. knowledge category) were examined using the chi‑square test of independence. Multivariable regression models were considered; however, given the descriptive nature of the study, the strong and consistent bivariate associations, and the presence of collinearity among several predictors, multivariable adjustment was not expected to provide additional explanatory value. Therefore, analyses focused on the primary bivariate associations.

## Results

The study encompassed a comprehensive survey of 3,420 individuals from the general population in the Qassim region, aged 18 and above, with 5.6% of the cohort being 50 years or older. A majority of the respondents were female, constituting 60.5% (2,068) of the sample. The predominant demographic was Saudi, accounting for 93.9% (3,210) (Table 1). 

**Table 1:** Demographic characteristics of the study population (N = 3420) Data are reported as frequencies and percentages.

Variable	Category	Frequency (n)	Percent
Age	18-34 years	2487	72.7
	35-49 years	740	21.6
	≥50 years	192	5.6
	Total	3420	100.0
Sex	Male	1352	39.5
	Female	2068	60.5
	Total	3420	100.0
Marital status	Married	1431	41.8
	Single	1989	58.2
	Total	3420	100.0
Educational level	School	569	16.6
	College	1949	57.0
	College graduate	651	19.0
	Postgraduate degree	251	7.3
	Total	3420	100.0

Regarding the prevalence of skin cancer risk factors, the majority of participants reported phenotypic and behavioural characteristics associated with increased susceptibility. More than half of the sample had Fitzpatrick skin types III-IV, and 23.4% reported having numerous moles. Daily sun exposure exceeding 1 hour was reported by 41.7% participants, and 18.2% indicated a history of frequent sunburns. Only a minority (6.4%) reported a family history of skin cancer. Collectively, these findings indicate that several modifiable and non‑modifiable risk factors were present within the study population, underscoring the relevance of targeted prevention strategies (Table 2).

**Table 2:** Risk of skin cancer among participants Risk‑related characteristics of the study population (N = 3420). Values are presented as frequencies and percentages for skin type, number of moles, daily sun exposure, and family history of skin cancer, as these variables represent descriptive risk indicators.

Variable	Category	Frequency (n)	Percent
Skin type	Fair (blonde/red hair, blue eyes, pale skin)	68	2.0
	Medium (brown/black hair, blue or brown eyes)	1351	39.5
	Dark (brown/black hair, brown eyes, dark skin)	2001	58.5
	Total	3420	100.0
Number of moles	0-5	2388	69.8
	5-10	845	24.7
	>20	187	5.5
	Total	3420	100.0
Daily sun exposure	<1 hour	2210	64.6
	1-2 hours	821	24.0
	3-4 hours	263	7.7
	>4 hours	126	3.7
	Total	3420	100.0
Family history of skin cancer	Yes	52	1.5
	No	3368	98.5
	Total	3420	100.0

Upon the application of a knowledge assessment, it was determined that 74.9% participants exhibited a commendable level of understanding, whereas 25.1% (858 individuals) demonstrated inadequate knowledge (Table 3).

**Table 3:** Assessment of participants' knowledge about skin cancer (N=3420) Values are presented as frequencies and percentages for each knowledge item and source of information.

Variable	Yes (n, %)	No (n, %)
Sun exposure can lead to skin cancer	1792 (52.4%)	1628 (47.6%)
Liability to skin cancer may be inherited	1102 (32.2%)	2318 (67.8%)
Skin cancer can be contagious	144 (4.2%)	3276 (95.8%)
Sun is more harmful to dark skin than fair skin	643 (18.8%)	2777 (81.2%)
Skin cancer can present with mucosal involvement	1383 (40.4%)	2037 (59.6%)
Skin cancer can be treated in early stages	3284 (96.0%)	136 (4.0%)
Chronic wounds can indicate skin cancer	343 (10.0%)	3077 (90.0%)
There is nothing to be done to protect from skin cancer	1661 (48.6%)	1759 (51.4%)
Source of Knowledge	Frequency (n)	Percent (%)
Doctor	270	7.9
Friends	137	4.0
Social media	1259	36.8
None	1754	51.3

The primary source of knowledge for participants, as reported by 36.8%, was social media. Moreover, we found that 94.1% (3218) of the participants expressed a favourable attitude towards skin cancer awareness and prevention. Most respondents agreed that using sunscreen, wearing protective clothing, and avoiding peak sunlight hours are important strategies for reducing skin cancer risk. However, fewer participants expressed confidence in their ability to perform skin self‑examinations or recognize early warning signs. These findings indicate that while preventive attitudes are favourable, uncertainty remains regarding early detection practices (Table 4).

**Table 4:** Participants' attitudes towards skin cancer and skin cancer prevention (N = 3420) Values are presented as frequencies and percentages across five‑point Likert‑scale responses.

Question	Strongly Agree (n, %)	Agree (n, %)	True Sometimes (n, %)	Strongly Disagree (n, %)	Disagree (n, %)
Skin is healthier when tanned	117 (3.4%)	116 (3.4%)	448 (13.1%)	1231 (36.0%)	1508 (44.1%)
I avoid prolonged exposure to sun for cosmetic reasons rather than medical reasons	436 (12.7%)	628 (18.4%)	766 (22.4%)	563 (16.5%)	1027 (30.0%)
Skin cancer is a dangerous, life‑threatening condition	737 (21.5%)	1050 (30.7%)	1075 (31.4%)	82 (2.4%)	476 (13.9%)
Sunscreen can be used for the protection of skin cancer	885 (25.9%)	1172 (34.3%)	896 (26.2%)	81 (2.4%)	386 (11.3%)
Usage of umbrella can help prevent skin cancer	156 (4.6%)	402 (11.8%)	811 (23.7%)	419 (12.3%)	1632 (47.7%)
Skin‑change observation is an important protective measure	1567 (45.8%)	1243 (36.3%)	507 (14.8%)	34 (1.0%)	69 (2.0%)

Educational level was significantly associated with participants’ knowledge status (χ² = 34.723, p < 0.001), with a small effect size (Cramér’s V = 0.10). Likewise, age group showed a statistically significant association with knowledge category (χ² = 53.41, p < 0.001), also demonstrating a small effect size (Cramér’s V = 0.13). These results indicate that although both age and educational level were significantly related to knowledge, the magnitude of these associations was modest.

## Discussion

Skin cancer remains a life‑threatening condition with a steadily increasing incidence worldwide. In the present study, we evaluated the general population’s knowledge and attitudes towards skin cancer, its risk factors, and preventive measures. The demographic characteristics of our sample indicate that the majority belonged to a relatively low‑risk group. Most participants were younger than 50 years of age, reported having fewer than five cutaneous moles, and only 2% exhibited phenotypic features associated with higher susceptibility, namely, fair, blonde, or red hair, blue eyes, and pale skin consistent with previously reported risk profiles [19]. The study identified several prevalent risk factors for skin cancer among adults in the Qassim region, including prolonged sun exposure, the presence of multiple moles, and a predominance of intermediate skin phototypes. Although the proportion of participants with a family history of skin cancer was low, the high frequency of behavioural risk factors, particularly extended daily sun exposure and prior sunburns, highlights the need for enhanced public‑health messaging on sun‑protective behaviours. These findings align with regional and international evidence showing that populations living in high‑UV environments often underestimate their personal risk. The presence of multiple risk factors in this population reinforces the importance of integrating risk‑factor education into community‑based awareness programs.

Participants were selected at random. Of the total, 78.3% of our participants were young adults (aged between 18 and 34 years), and 83.3% possessed university degrees or pursued postgraduate studies; this demographic factor may elucidate the commendable level of knowledge observed. Notably, 76% of participants were unaware that skin cancer could be hereditary. This finding may be attributed to the low incidence of skin cancer in Qassim, as evidenced by the fact that only 1% reported a family history of the disease. This lack of awareness may also account for more than half of the participants who were uninformed about the mucosal involvement associated with skin cancer. Mucosal involvement represents a critical clinical presentation for identifying potential skin cancer cases, as early recognition substantially improves survival outcomes, which are closely linked to disease stage and the presence or absence of metastasis [9,10,18]. Furthermore, our findings revealed that 90% of participants were unaware of the association between chronic, non‑healing wounds and the possible diagnosis of skin cancer. This highlights a significant gap in public awareness and underscores the urgent need for enhanced educational efforts regarding the diverse clinical manifestations of this malignancy. Beyond knowledge, participants’ attitudes towards preventive practices provide additional insight into the community’s readiness to engage in skin cancer prevention. Most respondents expressed positive attitudes towards sun‑protective behaviours, including the perceived importance of sunscreen use, protective clothing, and avoiding peak sunlight hours. However, fewer participants reported confidence in performing skin self‑examinations or identifying early warning signs. This pattern suggests that while preventive attitudes are favourable, practical knowledge related to early detection remains insufficient. Comparable studies in the region have reported similar discrepancies, where awareness of prevention does not consistently translate into confidence or competence in early recognition. These findings highlight the need for public health initiatives in the Qassim region that not only reinforce sun‑protective attitudes but also provide clear, accessible guidance on how to perform skin self‑examinations and recognize early signs of skin cancer.

Our findings of knowledge gaps were consistent with Basyouni et al. (2020), who conducted a similar study in Riyadh, which revealed a pervasive lack of knowledge and inadequate awareness among the general population [18].

In terms of knowledge sources, physicians were cited as the least-reported source by the enrolled general population (7.9%), in contrast to social media, which accounted for 36.8%. This aligns with the findings reported by Kelati et al. (2017), highlighting the considerably diminished role of physicians in the health education of the general population [14]. In contrast, a study conducted in India identified physicians as the primary source of skin cancer knowledge for the public, with 75% of participants reporting healthcare providers as their main source of information [16].

Furthermore, 34.3% of participants agreed that sun screening could play an effective role in skin cancer prevention. In contrast, Al Robaee’s study on the local Saudi population reported a markedly low rate of sunscreen use (8.3%) despite participants demonstrating a reasonably good understanding of the hazards associated with sun exposure [20].

In this study, both educational level and age demonstrated statistically significant associations with participants’ knowledge about skin cancer. However, the effect sizes for these relationships were small, indicating that although these demographic factors contributed to variations in knowledge, their overall influence was modest. The association between educational level and knowledge (Cramér’s V = 0.10) suggests that higher education may enhance awareness, but other unmeasured factors, such as access to health information, personal experiences, or exposure to public health campaigns, may also play important roles. Similarly, the small effect size observed for the age-knowledge association (Cramér’s V = 0.13) indicates that while older participants tended to demonstrate slightly better knowledge, age alone does not fully explain differences in awareness. These findings highlight the need for broad, population‑wide educational strategies rather than approaches targeted solely by age or educational attainment, consistent with the observations reported by Al Robaee and his research group [20]. We also identified statistically significant correlations between skin cancer awareness and age (p-value = 0.000), a pattern similarly noted by Alamri (p = 0.047) [12]. These results collectively suggest that educational interventions should prioritize individuals with lower levels of formal education, who may be at greater risk of inadequate awareness.

The study possesses notable strengths. It represents the first investigation conducted in the Qassim region to evaluate public awareness of skin cancer, incorporating a diverse sample drawn randomly from the general population to minimize selection bias. Moreover, the study included a substantially larger sample size compared to previous research carried out in Saudi Arabia and other Arabian Gulf countries, thereby enhancing the robustness and reliability of its findings.

It is important to acknowledge that the findings of this study cannot be generalized to the entire population of Saudi Arabia, and the application of an online survey can carry some potential biases. Additionally, skin cancer types were not differentiated; melanoma and BCC were collectively assessed under the broader category of skin cancer, which may limit the specificity of certain conclusions.

The online format of the questionnaire posed challenges in reaching certain high-risk populations, such as athletes, engineers, and construction workers. Future studies should focus on these at-risk groups. AlGhamdi et al. similarly discovered that protective clothing was the most frequently employed sun protection measure, reported by over 90% of the local Saudi population [21].

Furthermore, the Ministry of Labour and Social Development in Saudi Arabia has instituted and enforced regulations to ensure employee safety, prohibiting outdoor work from 12:00 PM to 3:00 PM from June to September when temperatures soar. This initiative is particularly commendable [22].

One of the weak points of this study was that skin cancer was not sub-classified into melanoma and non-melanoma types, which are both referred to as skin cancer. In addition, no systematic sampling technique was used, as participation was entirely voluntary. Although the questionnaire was adapted from previously validated tools and aligned with recommended guidelines for assessing skin cancer knowledge, tanning attitudes, or sun-related behaviours, there is currently no universally accepted gold‑standard instrument for evaluating skin cancer knowledge, tanning attitudes, or sun‑related behaviours. This limitation reduces the ability to directly compare our findings with those of other studies. Although the study provides valuable insights, several methodological limitations should be acknowledged. First, the use of a self‑administered questionnaire may have introduced social desirability bias, whereby participants could over-report positive attitudes or preventive behaviours related to skin cancer. This bias may have inflated the proportion of respondents reporting good knowledge or favourable attitudes, potentially leading to an overestimation of true awareness levels in the population. Second, the cross‑sectional design captures exposure and outcome simultaneously, which precludes establishing temporal or causal relationships, particularly regarding whether higher education leads to better knowledge or whether individuals with greater knowledge seek more education or information. Therefore, all associations identified should be interpreted as correlational rather than causal. These limitations have been acknowledged and considered when interpreting the findings.

## Conclusions

This study provides a comprehensive assessment of public awareness and attitudes towards skin cancer in the Qassim region. Although participants demonstrated generally good knowledge of risk factors, warning signs, and the importance of early detection, notable gaps persist, particularly regarding hereditary risk, mucosal involvement, and the association between chronic non‑healing wounds and skin cancer. While attitudes towards sun‑protective behaviours were largely positive, many respondents expressed limited confidence in performing skin self‑examinations or recognizing early suspicious lesions, indicating that practical knowledge remains insufficient despite favourable perceptions of prevention.

These findings underscore the need for targeted public health initiatives that extend beyond general awareness campaigns to include skill‑based education on early detection and self‑examination techniques. Strengthening community-level education, improving the role of healthcare providers in public counselling, and addressing misconceptions may enhance early recognition and reduce the burden of skin cancer in the region. Future research should focus on high‑risk occupational groups and incorporate more comprehensive assessment tools to further refine prevention strategies.

## Appendices

Questionnaire

Section 1 (demographics and general criteria of participating population)

1. Age

a. 18-34

b. 35-49

c. More than 50

2. Sex

a. Male

b. Female

3. Marital state

a. Single

b. Married

4. Educational level

a. School

b. College

c. Graduate

d. Postgraduate

Section 2 (Assessment of risk for skin cancer among participants)

1. Skin type

a. Fair-blonde or red hair with blue eyes and pale skin

b. Medium-brown or black hair and blue or brown eyes

c. Dark-brown or black hair, brown eyes, and dark skin

2. Number of moles

a. 0-5

b. 5-10

c. More than 20

3. Suration of daily sun exposure

a. Less than 1 hour

b. 1-2

c. 3-4

d. Less than 4

4. Do you have a member of your family as diagnosed as skin cancer patient

a. Yes

b. No

Section 3 (Knowledge about skin cancer)

1. Sun exposure can lead skin cancer

a. Yes

b. No

2. The liability to skin cancer can be inherited

a. Yes

b. No

3. skin cancer can be contagious

a. Yes

b. No

4. Sun is more harmful to dark skin than to fair skin

a. Yes

b. No

5. Skin cancer can present with mucosal involvement

a. Yes

b. No

6. Skin cancer can be treated in early stages

a. Yes

b. No

7. Chronic wounds can indicate skin cancer

a. Yes

b. No

8. There is nothing to be done for protection from skin cancer

a. Yes

b. No

9. Your source of knowledge information

a. Doctor

b. Friend

c. Media

D. None

Section 4 (Attitudes)

1. Skin is healthier when tanned

a. Strongly agree      b. Agree      c. Neutral     d. Disagree     e. Strongly disagree

2. I avoid prolonged exposure to sun for cosmetic reasons rather than medical reasons

a. Strongly agree      b. Agree      c. Neutral     d. Disagree     e. Strongly disagree

3. Skin cancer is a dangerous, life-threatening condition

a. Strongly agree      b. Agree      c. Neutral     d. Disagree     e. Strongly disagree

4. Sunscreen can be effective for protection of skin cancer

a. Strongly agree      b. Agree      c. Neutral     d. Disagree     e. Strongly disagree

5. Usage of umbrella can help as prevention of skin cancer

a. Strongly agree      b. Agree      c. Neutral     d. Disagree     e. Strongly disagree

6. Sunscreen usage can lead to vitamin D deficiency

a. Strongly agree      b. Agree      c. Neutral     d. Disagree     e. Strongly disagree

7. Skin changes observation is an important protective measure

a. Strongly agree      b. Agree      c. Neutral     d. Disagree     e. Strongly disagree

* Scoring and Classification

Knowledge Section: The knowledge scale consisted of nine items. Participants who answered fewer than five items correctly were classified as having poor knowledge, while those who answered five or more items correctly were classified as having good knowledge.

Attitudes Section: Attitude items were scored according to the direction of the statement. Participants were categorized as having positive or negative attitudes based on their total attitude score, with higher scores indicating more positive attitudes toward skin‑cancer prevention and awareness.
